# Moxibustion Inhibits the ERK Signaling Pathway and Intestinal Fibrosis in Rats with Crohn's Disease

**DOI:** 10.1155/2013/198282

**Published:** 2013-07-22

**Authors:** Xiaomei Wang, Yuan Lu, Luyi Wu, Chen Zhao, Chunbin Song, Shuguang Yu, Baixiao Zhao, Tianping Zhao, Huirong Liu, Chuanzi Dou, Yingying Zhang, Huangan Wu

**Affiliations:** ^1^Yueyang Hospital of Integrated Traditional Chinese and Western Medicine, Shanghai University of Traditional Chinese Medicine, Shanghai 200437, China; ^2^Key Laboratory of Acupuncture-Moxibustion and Immunological Effects, Shanghai University of Traditional Chinese Medicine, Shanghai 200030, China; ^3^Laboratory of Experimental Acupuncture of College of Acumox and Tuina, Chengdu University of Traditional Chinese Medicine, Chengdu 610075, China; ^4^School of Acupuncture-Moxibustion and Tuina, Beijing University of Chinese Medicine, Beijing 100029, China

## Abstract

Intestinal fibrosis is the main pathological process in Crohn's disease (CD); acupuncture and moxibustion can inhibit the process of fibrosis in CD rats, but the regulatory mechanism remains unknown. The present study observed the effect of moxibustion on the extracellular signal-regulated kinase (ERK) signaling pathway in the CD rat. The result shows that the phosphorylation of the Ras, Raf-1, MEK-1, and ERK-1/2 proteins and the expression of the corresponding mRNAs in the colon tissue of CD rat were significantly higher than the normal control group. Both treatments with mild moxibustion and with herb-separated moxibustion significantly reduced the expression of the Ras, Raf-1, MEK-1, and ERK-1/2 proteins and Ras and Raf-1 mRNA. MEK-1 and ERK-1/2 mRNA expression in each treatment group showed a downward trend, and the ERK-1/2 mRNA levels were significantly lower in the mild moxibustion group. It indicates that Ras, Raf-1, MEK-1, and ERK-1/2 are involved in the process of intestinal fibrosis in CD rats. Moxibustion can downregulate the abnormal expression of colonic Ras, Raf-1, MEK-1, and ERK-1/2 protein and mRNA levels in CD intestinal fibrosis in rats. Moxibustion may play a role in the treatment of CD intestinal fibrosis by regulating ERK signaling pathway.

## 1. Introduction 

Crohn's disease (CD) is an agnogenic, chronic, and nonspecific granulomatous inflammatory disease of the intestine [1]. The histological features of CD are characterized by transmural inflammation, lymphangiectasia, and lymphatic and fibrous tissue hyperplasia. CD is more common in Europe and the USA than in other countries. The annual incidence in Europe and the USA is 5–10 per 100,000 persons, and the prevalence is 50–100 per 100,000 persons [2]. The incidence of CD in Asia is lower than in Western countries, although the incidence in China has noticeably increased in recent years [3, 4].

Under the pathological conditions of the chronic and recurring inflammation of CD, collagen decomposition is limited, and the continuously increasing deposition of collagen fibers results in excessive scar tissue, which promotes the hardening of the intestinal wall and luminal stenosis. Therefore, local intestinal function is impaired [5]. The results of impaired intestinal function range from the loss of intestinal wall compliance, severe abdominal pain, anorexia, and diarrhea to priming fistula formation and even increasing the possibility of malignant transformation, in which disease duration is persistent and refractory [6]. Studies have shown [7, 8] that early mild intestinal fibrosis can be reversed, which has great significance for slowing disease. At present, the basis of therapy for CD intestinal fibrosis is long-term anti-inflammatory treatment. However, although they may reduce inflammation, anti-inflammatory treatments do not reduce fibrous stenosis. Therefore, an understanding of the pathogenesis of CD intestinal fibrosis and the discovery of effective prophylactic measures with fewer side effects are urgently needed in this field.

Intestinal fibrosis is a complicated pathological process in which many cell types, cytokines, and signaling pathways participate, including the extracellular signal-regulated kinase (ERK) signaling pathway, which has become a popular topic of current study. ERK, including ERK1 and ERK2, is one of the mitogen-activated protein kinase (MAPK) signaling pathways. The ERK1/2-MAPK signaling pathway is a cascade that is related to the pathological fibrosis of multiple diseases [9]. An activated Ras-Raf-MEK-ERK pathway promotes fibrosis and plays an important role in TGF-*β*-mediated type I collagen expression, extracellular matrix (ECM) production, and myofibroblast formation [10, 11].

Preliminary studies have shown that medicine-separated moxibustion can significantly inhibit the process of fibrosis in CD rats [12, 13], but the regulatory mechanism underlying this process remains to be further explored. In this study, a CD intestinal fibrosis rat model was prepared by the Morris method [14]. EnVision immunohistochemistry and qPCR were used to measure the levels of Ras, Raf, MEK, and ERK proteins and mRNA expression in colon tissues to explore the key signaling molecules involved in the regulation of CD intestinal fibrosis in rats and reveal the mechanisms underlying the effects of moxibustion on CD intestinal fibrosis.

## 2. Materials and Methods

### 2.1. Experimental Animals

A total of 60 male Sprague-Dawley (SD) rats with a body weight of 180 ± 20 g were provided by the Experimental Animal Center at the Shanghai University of Traditional Chinese Medicine. All of the rats were kept for one week at room temperature after receipt. The rats that behaved normally with respect to feeding, drinking, and other behaviors were included in the experiment. This study received permission from the Ethics Committee in Yueyang Hospital, which is affiliated with Shanghai University of Traditional Chinese Medicine, China. All protocols were performed in strict accordance with *the Guidance Suggestions for the Care and Use of Laboratory Animals*, formulated by the Ministry of Science and Technology of the People's Republic of China [15].

### 2.2. Grouping and Model Establishment

A total of 60 SD rats were randomly divided into a normal group of 12 rats and a model group of 48 rats. A 2,4,6-trinitrobenzene sulfonic acid (TNBS) (Sigma Co., St. Louis, USA) enema of 5% TNBS and 50% ethanol was prepared at a 2 : 1 ratio. The rats in each group were fasted but not deprived of water for 48 h. The rats in the model group were anesthetized by the intraperitoneal injection of 2% sodium pentobarbital (China Langchem Inc., Shanghai, China) at a dose of 20 mg/kg and were vertically inverted to introduce 100 mg/kg TNBS by rectal infusion once per 7 days for a total of four treatments, whereas the normal group was not treated. To validate the model, the distal colons from one rat in the normal group and two rats in the model group were examined by visual observation and H&E staining. After the success of the model was confirmed, the 44 rats were randomly assigned to a model group, a mild moxibustion group, a herb-separated moxibustion group, and an EA group, with 11 rats per group. Two rats died during the modeling process, and one rat from the model group and one rat from the mild moxibustion group died during the course of treatment.

### 2.3. Treatment****

*NC Group*. No treatment.

*MC Group*. TNBS enema only, no treatment.

*MM Group*. A TNBS enema was administered, and the ST25 (bilateral) and RN6 acupuncture points were located. A moxa stick (Nanyang Hanyi Moxa Co., Ltd. China) 5 mm in diameter was lit and placed at points at a distance of 1-2 cm from the ST25 and RN6 acupuncture points. Moxibustion was performed for 10 min per point per treatment, once a day for a total of seven treatments.

*HSP Group*. A TNBS enema was administered, and the ST25 (bilateral) and RN6 acupuncture points were located. Aconite, cinnamon, salvia, and other drugs were milled into a powder and mixed with rice wine to form a paste. The paste was then pressed into a medicinal cake, 5 mm in diameter and 3 mm in thickness. A moxa cone was formed from 90 mg of refined moxa. Two moxa cones were used for moxibustion for each point per day, and a total of seven moxibustion treatments were performed for 7 days.

*EA Group*. A TNBS enema was administered, and the ST25 (bilateral) and RN6 acupuncture points were located. Needles (Suzhou Huatuo Co., Ltd. Suzhou, China) 0.25 mm in diameter and 25 mm in length were inserted to a depth of 3-4 mm and connected to an LD202H Han electroacupuncture apparatus (Beijing Huawei Co., Ltd. Beijing, China). The RN6 and bilateral ST25 acupuncture points were stimulated alternately by a continuous wave of 0.3 ms pulse width, 100 Hz frequency, and 1 mA current once a day for 10 min, with continuous treatment for 7 days.

### 2.4. Specimen Collection

Following treatment, the rats were fasted for 24 h with access to water and were then anesthetized by intraperitoneal injection of 2% sodium pentobarbital at a dose of 50 mg/kg. The anesthetized rats were then surgically sectioned to remove approximately 6 cm of the distal colon. The mesenterium was then cut longitudinally and rinsed with 4°C saline. The mucosal injury was visually examined and evaluated using a gross injury score [16, 17]. Two pieces of tissue with obvious lesions were removed, of which one was transferred to liquid nitrogen until testing, and the other was fixed in a 10% neutral formaldehyde solution.

### 2.5. Detection of Collagen Fibers in the Rat Colon

Masson trichrome staining was used to detect collagen fibers in the rat colon. Green staining was interpreted as collagen, mucus, and cartilage; red staining denotes muscle and nerve fibers; and black indicates nuclei.

### 2.6. EnVision Plus Immunohistochemical Detection of Ras, Raf, MEK, and ERK-1/2 in Colonic Tissue

Fixed tissues were conventionally deparaffinized in water and washed three times for 3 min in 0.01 M PBS, pH 7.4. The tissue samples were heated and allowed to equilibrate to room temperature for 20 min and then washed three times in PBS for 3 min. To inhibit endogenous peroxidase, the samples were treated with 0.3% H_2_O_2_ at room temperature for 20 min and then rinsed three times with PBS for 3 min. The tissue samples were incubated in 20% normal goat serum at room temperature for 30 min and then with one drop of the appropriately diluted primary antibody (Santa Cruz Bio Inc., Santa Cruz, USA) at 4°C for 18 h. The tissues were washed three times with PBS for 3 min, mixed with one drop of Envision reagent (HRP-M), and incubated in a humidified chamber for 30 min at 37°C. After washing three times with PBS for 3 min, the assay was developed in 0.04% DAB and 0.3% H_2_O_2_ for 8–12 min, then washed in water, stained with hematoxylin for 1 min, and bathed in hot water for 3 sec to allow the blue color to develop. Finally, the tissue sections were dried and mounted with a neutral resin. All the samples were analyzed by a Motic Med 6.0 image analysis system (Motic Group Co., Ltd.). Three fields were randomly selected, the positive target value of the integral optical density was calculated under an optical microscope (Olympus Co., Ltd.) at 200x magnification.

### 2.7. qPCR Detection of Ras, Raf, MEK, and ERK-1/2 mRNA Levels in Colonic Tissues

The total RNA was extracted from colon tissue samples. The cDNA was synthesized by reverse transcription (RT) according to the qPCR kit (Shanghai Daweike Bio Inc., Shanghai, China) instructions and amplified by PCR according to the kit instructions (Table 1). The following cycling conditions were used: RT at 37°C for 1 h, 95°C for 5 min to inactivate the MMLV RT enzyme, 50°C for 2 min, 95°C for 5 min, and 40 cycles of 95°C for 15 sec and 60°C for 45 sec.

### 2.8. Statistical Analysis

SPSS 18.0 statistical software (SPSS Inc., Chicago, USA) was used for statistical analysis. The measurement data are presented as the mean ± SD. For normally distributed data, one-way analysis of variance (ANOVA) was applied. The least significant difference method (LSD) was used for the homogeneity of variance, and Games-Howell was used to compare the differences between groups for the heterogeneity of variance; A value of *P* < 0.05 was considered statistically significant. The nonparametric Mann-Whitney *U*-test was used for abnormal distributions.

## 3. Results

### 3.1. Gross Injury in the Colon Tissue of Rat

When the colonic tissues were observed by visual inspection, the rats in the normal group exhibited a uniformity of colon bowel and intestinal wall, a smooth colonic mucosa surface, clear vascular texture, no bleeder, and no erosion or ulcers. The model group exhibited partial intestinal canal stenosis or intestinal wall thicken and harden, fuzzy vascular texture, edematous colonic mucosa, serious congestion, erosion, and different size, shape, and depth ulcer formation. After treatments, there were lacked smooth colonic mucosa surface, edematous colonic mucosa and erosion in both MM and EA group, and lacked smooth colonic mucosa surface with no obvious erosion and ulcer in HPM group.

**Figure 1:**
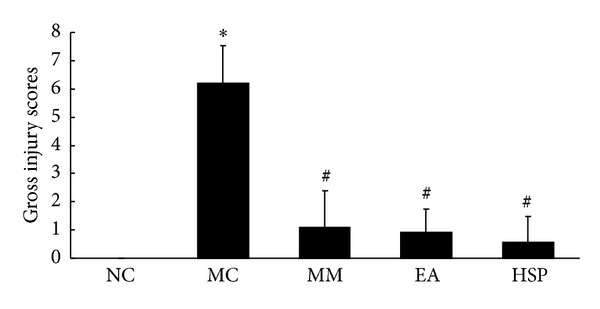
The gross injury scores of the colon tissue in each rat group. NC: normal control group, MC: model control group, MM: mild moxibustion, EA: electroacupuncture, and HSM: herb-separated moxibustion. *P < 0.01 versus NC; ^#^ *P* < 0.01 versus MC.

As shown in Figure 1, the gross injury score of the colonic tissue in the NC group was significantly higher than the MC group (*P* < 0.01). After treatment, the scores were lower in both the HPM group, MM group, and the EA group (*P* < 0.01). It suggests that these treatments are able to improve the tissue damage of CD. 

### 3.2. Expression of Collagen Fibers in the Colon Tissue of Rat

**Figure 2:**
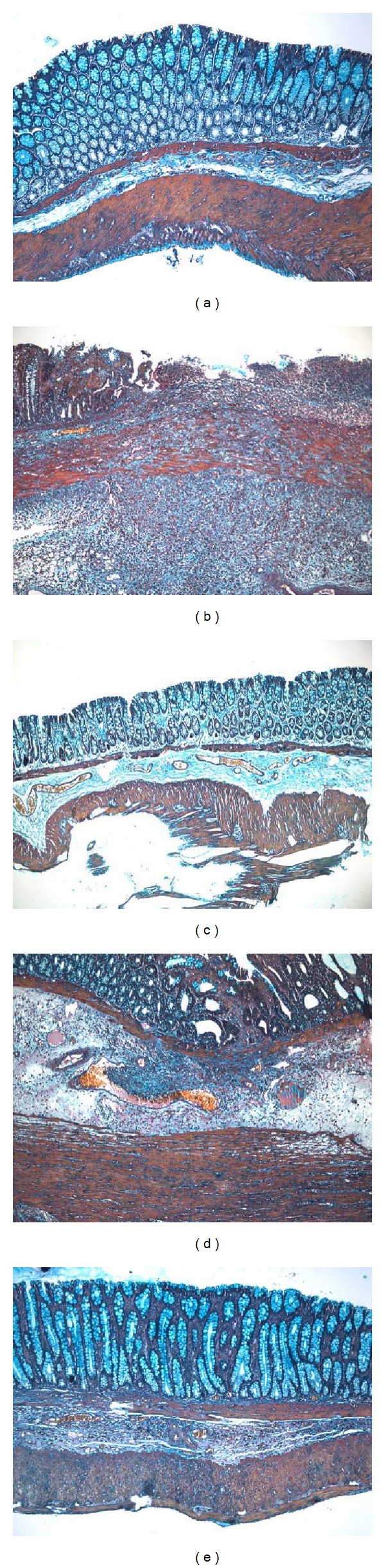
Expression of collagen fibers in the colon tissue of rat. Masson trichrome staining method, ×100. (a) Normal; (b) Crohn's disease; (c) mild moxibustion; (d) electroacupuncture; (e) herb-separated moxibustion.

As shown in Figure 2, Masson trichrome staining revealed that in the MC group, a large number of collagen fibers were present in the mucosa, muscularis mucosa, submucosa, and muscularis of the rat colon, with a disordered arrangement. In the MM group and the HSM group, there were small numbers of collagen fibers in the colonic lamina propria and submucosa at the original ulcer-healing location, which was less severe compared with that of the model group. In the EA group, there were a small number of collagen fibers in the mucosa, submucosa, muscularis, and serosa of the rat colon, and the effect was less severe than in the MC group.

### 3.3. Ras Expression in the Colonic Mucosa of Rat

**Figure 3:**
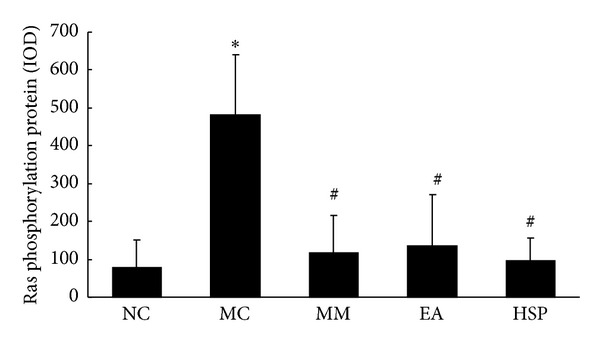
The integral optical density (IOD) of Ras phosphorylation in each rat group. NC: normal control group, MC: model control group, MM: mild moxibustion, EA: electroacupuncture, and HSM: herb-separated moxibustion. *P < 0.01 versus NC; ^#^ *P* < 0.01 versus MC.

**Figure 4:**
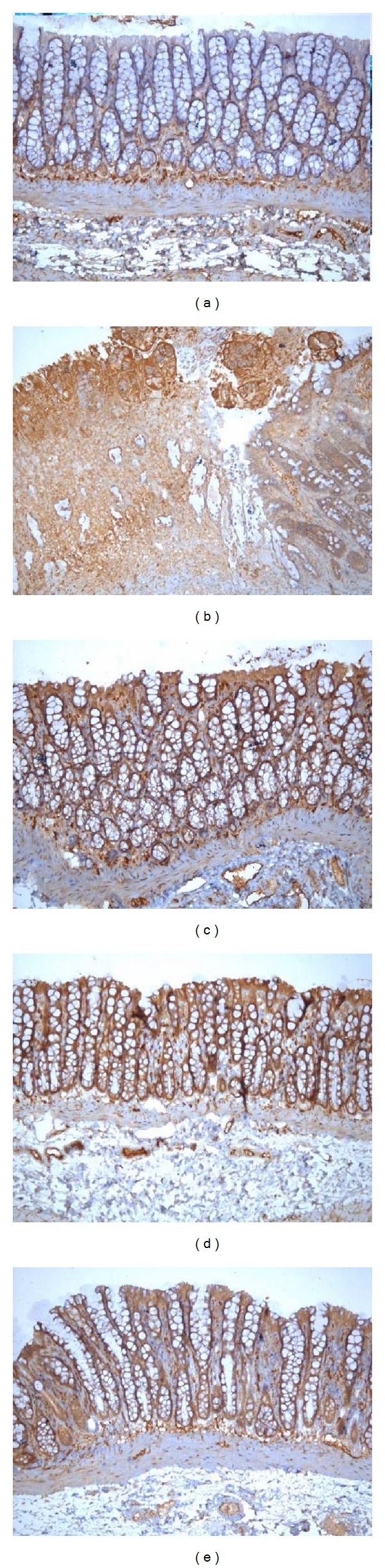
Expression of phosphorylated Ras protein in the colonic mucosa of rat. The phosphorylation of Ras protein was expressed in epithelial cells and lymphocyte in the colonic mucosa of rats. EnVision Plus method, ×200. (a) Normal; (b) Crohn's disease; (c) mild moxibustion; (d) electroacupuncture; (e) herb-separated moxibustion.

As shown in Figures 3 and 4, the phosphorylation of Ras protein in the rat colon was significantly higher in the MC group than in the NC group (*P* < 0.01). Ras protein expression was significantly reduced (*P* < 0.01) to normal levels after acupuncture treatment.

**Figure 5:**
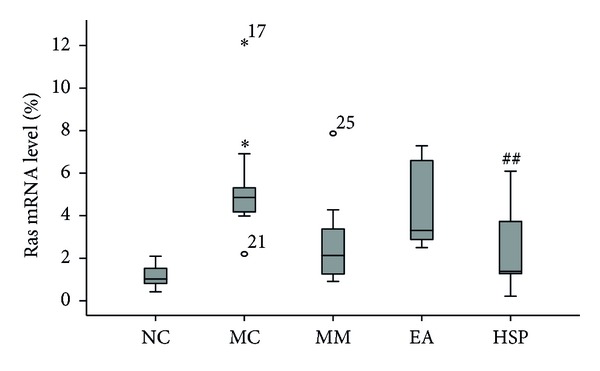
The Ras mRNA levels in the rat colon. NC: normal control group, MC: model control group, MM: mild moxibustion, EA: electroacupuncture, and HSM: herb-separated moxibustion. *P < 0.01 versus NC; ^##^ *P* < 0.05 versus MC.

Figure 5 indicates that Ras mRNA expression in the MC group was higher than that in the NC group (*P* < 0.01) and that HSM effectively reduced the Ras mRNA levels in the rat colon (*P* < 0.05), whereas there was no significant difference between the EA group and the NC group. 

### 3.4. The Raf-1 Expression in the Colonic Mucosa of Rat

**Figure 6:**
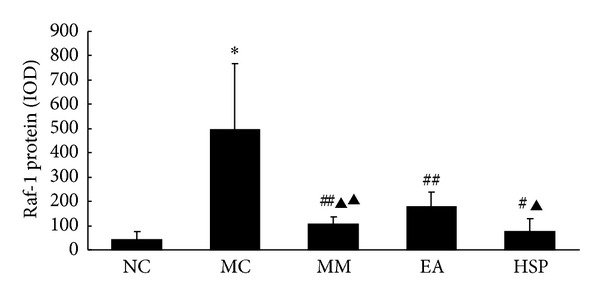
The IOD of Raf-1 protein expression in rat colon. NC: normal control group, MC: model control group, MM: mild moxibustion, EA: electroacupuncture, and HSM: herb-separated moxibustion. *P < 0.01 versus NC; ^##^ *P* < 0.05, ^#^ *P* < 0.01 versus MC; ^▲▲^ *P* < 0.05, ^▲^ *P* < 0.01 versus EA.

**Figure 7:**
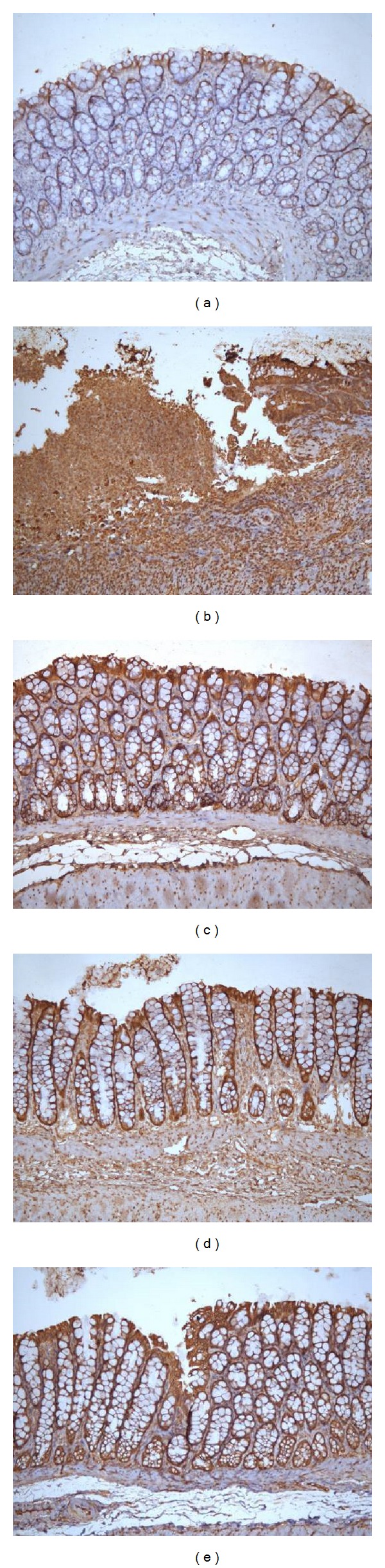
Expression of Raf-1 protein in the colonic mucosa of rat. The Raf-1 protein was expressed in epithelial cells in the colonic mucosa of rats. EnVision Plus method, ×200. (a) Normal; (b) Crohn's disease; (c) mild moxibustion; (d) electroacupuncture; and (e) herb-separated moxibustion.

As shown in Figures 6 and 7, the Raf-1 protein expression in the colonic tissue of the MC group was significantly higher than in the NC group (*P* < 0.01) and that the Raf-1 protein expression in the colon was reduced to varying degrees after acupuncture treatment (*P* < 0.05 or *P* < 0.05).

**Figure 8:**
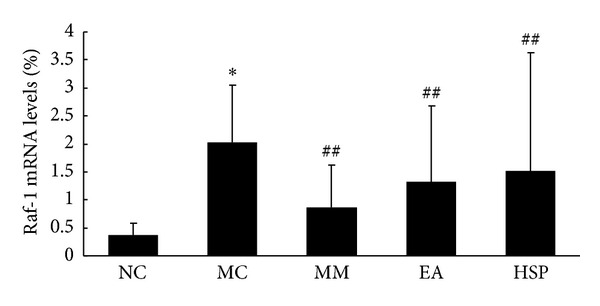
The Raf-1 mRNA levels in the rat colon. NC: normal control group, MC: model control group, MM: mild moxibustion, EA: electroacupuncture, and HSM: herb-separated moxibustion. *P < 0.01 versus NC; ^##^ *P* < 0.05 versus MC.

The results in Figure 8 show that the Raf-1 mRNA levels in the MC group were significantly higher than in the NC group (*P* < 0.01) and that MM and HSM treatments significantly reduced rat colon Raf-1 mRNA levels (*P* < 0.05), whereas EA treatment did not significantly affect rat colon Raf-1 mRNA levels.

### 3.5. The MEK-1 Expression in the Colonic Mucosa of Rat

**Figure 9:**
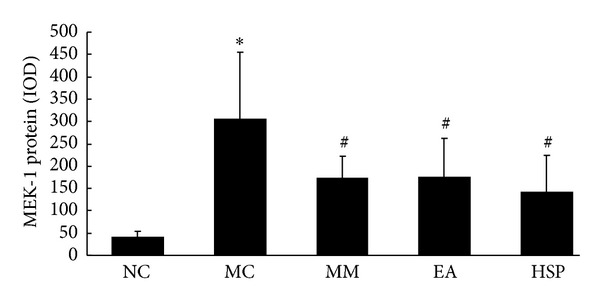
The IOD of MEK-1 protein expression in the rat colon. NC: normal control group, MC: model control group, MM: mild moxibustion, EA: electroacupuncture, and HSM: herb-separated moxibustion. *P < 0.01 versus NC; ^#^ *P* < 0.01 versus MC.

**Figure 10:**
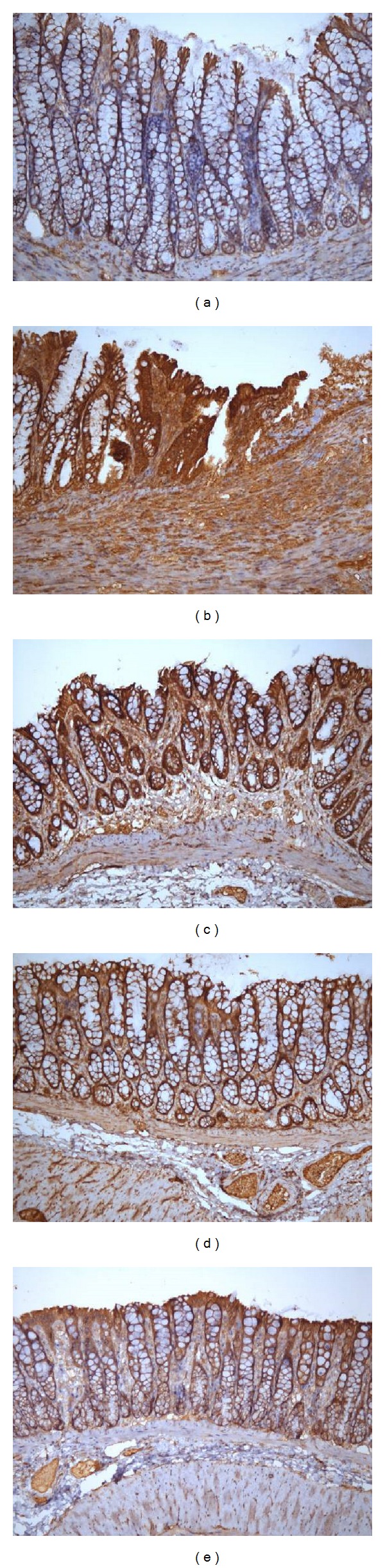
Expression of MEK-1 protein in the colonic mucosa of rat. The MEK-1 protein was expressed in epithelial cells in the colonic mucosa of rats. EnVision Plus method, ×200. (a) Normal; (b) Crohn's disease; (c) mild moxibustion; (d) electroacupuncture; and (e) herb-separated moxibustion.

As shown in Figures 9 and 10, the MEK-1 protein expression in the colonic tissue of the MC group was significantly higher than in NC rats (*P* < 0.01), and MEK-1 protein expression was significantly reduced in each group after acupuncture treatment (*P* < 0.01), although the levels were still higher than normal levels (*P* < 0.01).

**Figure 11:**
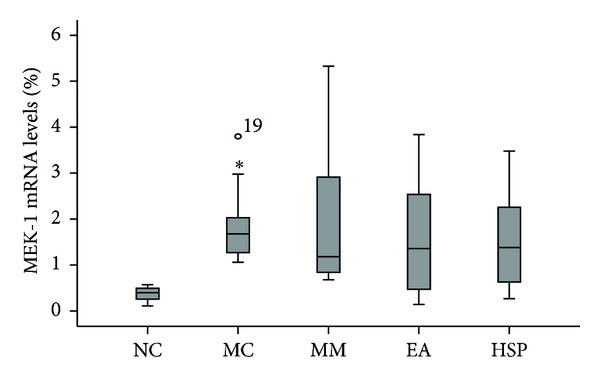
The MEK-1 mRNA levels in the rat colon. NC: normal control group, MC: model control group, MM: mild moxibustion, EA: electroacupuncture, and HSM: herb-separated moxibustion. *P < 0.01 versus NC.

The results in Figure 11 show that the MEK-1 mRNA levels in the model group were significantly higher than those in the NC group (*P* < 0.01) and that MM, HSM, and EA treatment reduced MEK-1 mRNA levels, although there was no statistically significant difference between the treatment and no treatment groups or among the treatment groups.

### 3.6. The ERK-1/2 Expression in the Colonic Mucosa of Rat

**Figure 12:**
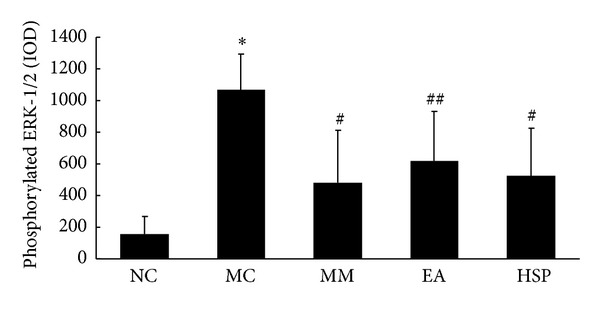
The IOD of phosphorylated ERK-1/2 levels in the rat colon. NC: normal control group, MC: model control group, MM: mild moxibustion, EA: electroacupuncture, and HSM: herb-separated moxibustion. *P < 0.01 versus NC; ^##^ *P* < 0.05, ^#^ *P* < 0.01 versus MC.

**Figure 13:**
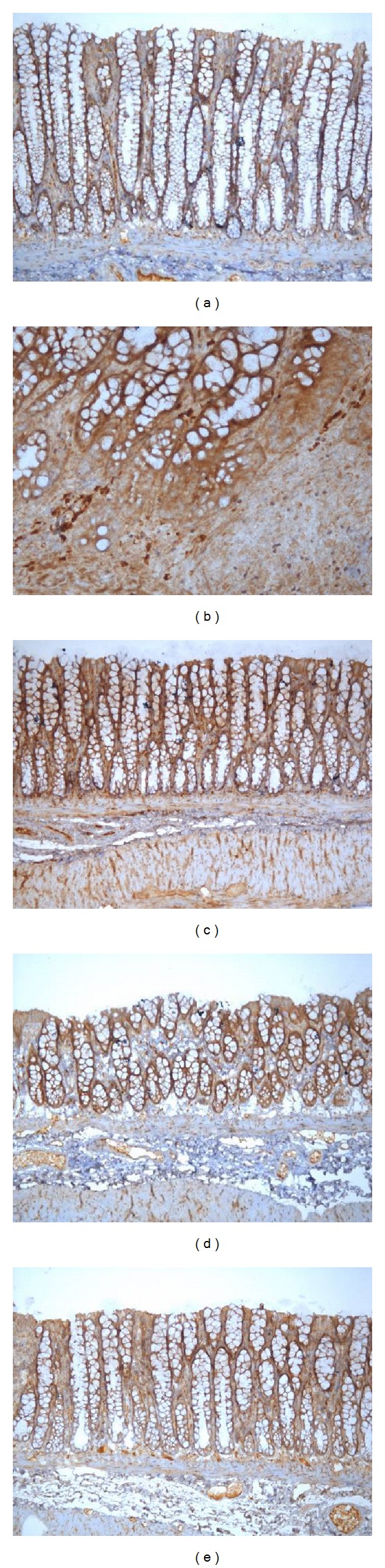
Expression of phosphorylated ERK-1/2 protein in the colonic mucosa of rat. The phosphorylation of ERK-1/2 protein was expressed in epithelial cells and lymphocyte in the colonic mucosa of rats. EnVision Plus method, ×200. (a) Normal; (b) Crohn's disease; (c) mild moxibustion; (d) electroacupuncture; and (e) herb-separated moxibustion.

As shown in Figures 12 and 13, the phosphorylation of ERK-1/2 significantly increased in the MC group compared with the NC group (*P* < 0.01) and that the levels of phosphorylated ERK-1/2 proteins were significantly reduced after the MM and HSM treatments (*P* < 0.01).

**Figure 14:**
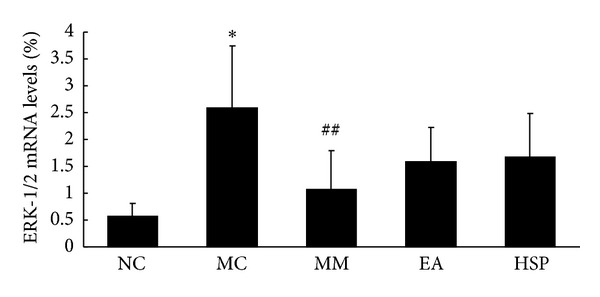
The ERK-1/2 mRNA levels in the rat colon. NC: normal control group, MC: model control group, MM: mild moxibustion, EA: electroacupuncture, and HSM: herb-separated moxibustion. *P < 0.01 versus NC; ^##^ *P* < 0.05 versus MC.

The results in Figure 14 show that the ERK-1/2 mRNA expression in the MC group was significantly higher than that in the NC group (*P* < 0.01) and that ERK-1/2 mRNA expression was significantly lower in the MM group after acupuncture treatment compared with the MC group (*P* < 0.05).

## 4. Discussion

Extracellular signal-regulated kinase (ERK), a member of the mitogen-activated protein kinase (MAPK) family, plays an important biological role in transmitting signals from surface receptors to the nucleus. The ERK1/2 pathway is the most thoroughly researched pathway in the ERK family and plays a role in a wide range of cellular functions, including regulating meiosis, mitosis, and anaphase processes in different cells. A variety of stimulating factors, such as growth factors, cytokines, viruses, G protein-coupled receptor ligands, and oncogenes, can activate the ERK1/2 pathway [18]. The ERK signaling pathway is a Ras-Raf-MEK-ERK pathway. Phosphorylation-activated ERK1/2 translocates from the cytoplasm to the nucleus and thereby mediates the transcriptional activation of Elk-1, ATF, NF-*κ*B, AP-1, and c-fos, which in turn regulate cell proliferation and differentiation, the maintenance of cell shape, the construction of the cytoskeleton, apoptosis, and the malignant transformation of cells, among other biological functions.

One of the features of CD is the transformation of smooth muscle cells into fibroblasts and/or myofibroblasts [8]. This abundance of fibroblasts and myofibroblasts increases the expression of type I collagen mRNA and produces collagen tissue within the muscle [19]. There are a large number of fibroblast cells and type I, III, IV, and V collagen transcripts in the small intestines of CD patients [20], which produce more ECM, including collagen, elastin, and glycoproteins that are deposited intramurally to produce a thickening of the intestinal wall [10]. Based on a large number of experimental reports, the ERK-1/2 signaling pathway appears to participate in myofibroblast proliferation, differentiation, and collagen production [10, 11, 21–25], extracellular matrix synthesis [26, 27], smooth muscle cell proliferation and apoptosis [28], and the migration of vascular smooth muscle cells [29]. Inhibiting the expression and activity of ERK can inhibit the proliferation of vascular smooth muscle cells [30] to reduce the accumulation of ECM [31]. Therefore, it is inferred that the ERK-1/2 signaling pathway may induce the activation and proliferation of CD intestinal myofibroblasts and their transformation into muscle fiber cells. Additionally, the ERK-1/2 signaling pathway may regulate the proliferation and apoptosis of smooth muscle cells.

As an upstream protein in the Raf-MEK-ERK pathway, Ras is the product of the proto-oncogene *c-ras*. A variety of stimulating factors, particularly growth factors (e.g., PDGF, EGF, TNF, and PKC activators as well as Src family members) can activate Ras, which further activates Raf-1. Activated Raf-1 then activates MEK and ERK downstream [32, 33] to initiate the MAPK pathway. Raf-1 protein kinase is widely expressed in various types of cells [34]. Ras and Raf-1 often exist in the form of complexes. Ras, the upstream activator protein, can translocate Raf from the cytoplasm to the plasma membrane, where the latter is activated. However, the mechanism by which Raf is activated remains unclear, although it may include phosphorylation [35]. All that is known is that Raf activation is related to its phosphorylation on Ser/Thr residues [33]. Recent studies have shown that Raf-1 protein kinase may be a point of crosstalk with other signaling pathways. This protein may participate in a variety of biological processes in signal transduction, including its own regulation, by interacting with other signaling molecules or via reciprocal regulation. 

The MAPK kinases include MEK1 (MKK1) and MEK2 (MKK2). MEK1 and MEK2 are the central links in the MAPK pathway. The activated upstream Raf-1 protein kinase can combine with MEK1 and 2 and doubly phosphorylate Tyr and Thr, its two regulatory sites, to activate ERK [36]. MEKs are not only ERK activators but may also relocalize the activated ERKs to the nucleus or other active sites to further phosphorylate downstream substrates [37].

The upstream kinase MEK1 is a dual-specificity enzyme. Activated MEK1 can phosphorylate ERK1 and ERK2. There is evidence that the Tyr residues of ERK1 and 2 are phosphorylated before the Thr residues, both intracellularly and extracellularly [38]. ERK translocates into the nucleus by forming a homodimer of phosphorylated ERK and phosphorylated or unphosphorylated ERK [39]. ERK is evenly distributed in quiescent cells, but when the cells are stimulated by serum, persistently activated ERK can partially translocate into the nucleus, whereas transiently activated ERK cannot relocalize. Activated ERKs accumulate in the nucleus and then phosphorylate the corresponding transcription factors, which drive expression of specific gene products. Because of the difference in nuclear localization, transiently activated (or low-activity) ERK and persistently activated (or high-activity) ERK cause cells to express genes at a different quality or quantity, resulting in different biological effects [40–43].

The ERK1-ERK2-MAPK pathway incorporates a mechanism for inactivation after activation, which can prevent excessive activation-induced adverse consequences. At the same time, the inactivated kinase is prepared for the next activation. The mechanisms of inhibition of this pathway include the following: (1) the activation of ERK1/2-MAPK is the result of the phosphorylation cascade and can be inactivated by dephosphorylation by the corresponding phosphatases; (2) Raf-1 is the direct substrate of PKA, which can phosphorylate Ser43 of c-Raf, an inhibitory phosphorylation site in the N-terminus of Raf that interacts with Ras, and the phosphorylation of this site can inhibit the binding between RasGTP and the N-terminus of Raf, which blocks the activation of ERK; (3) the phosphatase PP2 can inactivate MEK and ERK via dephosphorylation; (4) activated ERK phosphorylates Sos and Raf while ERK dephosphorylation inactivates them, thereby generates negative feedback regulation of the pathway. Therefore, the study of the mechanism by which acupuncture regulates the expression of ERK upstream receptors, the phosphorylation of ERK cascade members, the targets of downstream signal transduction, and the potential impact of other signaling pathways on this pathway is necessary to confirm acupuncture as an antifibrotic treatment.

Our experiments studied the regulation of Ras, Raf, MEK, and ERK protein and gene expression by acupuncture in CD intestinal fibrosis in the rat colon. Our results show that the mRNA levels of Ras, Raf-1, MEK-1, and ERK-1/2 were significantly increased in CD rat intestinal tissue and that the expression of p-Ras, Raf-1, MEK-1, and p-ERK-1/2 proteins in intestinal epithelial cells were also significantly increased. These results suggest that the activity of the ERK pathway in CD intestinal fibrosis process was altered. Medicine-separated moxibustion mild moxibustion, and electroacupuncture treatments could effectively downregulate the expression of p-Ras, Raf-1, MEK-1, and p-ERK-1/2, but different inhibitory effects on Ras, Raf-1, MEK-1, and ERK-1/2 mRNA levels were shown in ERK pathway activity changes. In this discussion, we propose the following: (1) medicine-separated moxibustion, mild moxibustion, and electroacupuncture can inhibit CD intestinal fibrosis by regulating ERK pathway activity; and (2) the acupuncture-induced inhibition of p-Ras, Raf-1, MEK-1, and p-ERK-1/2 expression may be the key elements in the treatment's inhibition of CD intestinal fibrosis. At present, most reports regarding the role of acupuncture in the regulation of the ERK signaling pathway have focused on EA, such as EA model rats. After EA on the Dazhui (DU14) and Baihui (DU20) acupuncture points, the number of ERK-positive striatal cells increased significantly in the cerebral cortex, and ERK protein expression was increased in rats with damaged neurons after cerebral ischemia and reperfusion; therefore, EA can increase ERK pathway activity [44, 45]. After the EA treatment of gastric mucosa-injured rats, the phosphorylation levels of ERK in gastric mucosal cells from extracted serum were significantly increased and were significantly different between the serum of the normal rats and that of the model rats. These observations suggest that ERK may be involved in signal transduction in the EA stimulation at acupoints of stomach meridian of foot yangming, repairing gastric mucosal injury [46], and that EA had an analgesic effect by downregulating p-ERK expression levels in the dorsal horn of the spinal cord of rats with adjuvant arthritis [47]. The previous reports have demonstrated that EA can regulate the ERK signal transduction pathway. The results of our experiments show that medicine-separated moxibustion and mild moxibustion can also regulate the ERK signal transduction pathway and inhibit Ras and Raf-1 better than EA. The functional mechanisms underlying these observations need to be explored further.

## Figures and Tables

**Table 1:** The sequence of the forward primer and reverse primer used for fluorescent quantitative PCR assays.

Name	Primer sense	Sequence (5′→3′)	Amplification product (bp)
GAPDH	Forward	5′-CCGAGGGCCCACTAAAGG-3′	116 bp
	Reverse	5′-GCTGTTGAAGTCACAGGAGACAA-3′	
Ras	Forward	5′-TGGTGGGCAACAAGTGTGAC-3′	181 bp
	Reverse	5′-GGGTTCAGTTTCCGCAGTTTATG-3′	
Raf-1	Forward	5′-CAGGAGCACAAAGGTAAGAAAGC-3′	201 bp
	Reverse	5′-GCCACAAGTCTGACATCGAAATC-3′	
MEK-1	Forward	5′-AATATGTCAGGTTCAAGGAAAGGAC-3′	169 bp
	Reverse	5′-AGGTAAGGACGAAGCCACAAG-3′	
ERK-1/2	Forward	5′-TATATACATTCAGCTAATGTTCTGC-3′	169 bp
	Reverse	5′-TCTACCTGCAGCTGGAACCTCTC-3′	

## Abbreviations

CD: Crohn's disease
NC: Normal control group
MC: Model control group
EA: Electroacupuncture
MM: Mild moxibustion
HSM: Herb-separated moxibustion
ST25: Tianshu acupoint
RN6: Qihai acupoint
ERK: Extracellular signal-regulated kinase
MAPK: Mitogen-activated protein kinase
TNBS: 2,4,6-Trinitrobenzene sulfonic acid.
